# Activated sludge bacterial communities of typical wastewater treatment plants: distinct genera identification and metabolic potential differential analysis

**DOI:** 10.1186/s13568-018-0714-0

**Published:** 2018-11-14

**Authors:** Bo Zhang, Xiangyang Xu, Liang Zhu

**Affiliations:** 1 Department of Environmental Engineering, Zhejiang University, Hangzhou, 310058 People’s Republic of China; 2 Zhejiang Province Key Laboratory for Water Pollution Control and Environmental Safety, Hangzhou, 310058 China

**Keywords:** Activated sludge, High throughput sequence, Textile dyeing wastewater, Metagenomic, Metabolic potential

## Abstract

To investigate the differences in activated sludge microbial communities of different wastewater treatment plants (WWTPs) and understand their metabolic potentials, we sampled sludge from every biological treatment unit of 5 full-scale waste water treatment systems in 3 typical Chinese municipal WWTPs. The microbial communities and overall metabolic patterns were not only affected by influent characteristics but also varied between different biological treatment units. Distinct genera in different wastewater treatment systems were identified. The important microorganisms in domestic sewage treatment systems were unclassified *SHA*-*20*, *Caldilinea*, *Dechloromonas*, and unclassified genera from *Rhodospirilaceae* and *Caldilineaceae*. The important microorganisms in dyeing wastewater treatment systems were *Nitrospira*, *Sphingobacteriales*, *Thiobacillus*, *Sinobacteraceae* and *Comamonadaceae*. Compared with the obvious differences in microbial community composition, the metabolic potential showed no significant differences.

**Electronic supplementary material:**

The online version of this article (10.1186/s13568-018-0714-0) contains supplementary material, which is available to authorized users.

## Introduction

Textile dyeing and fine chemical industries have a prominent focus in certain areas of eastern China. From these, a large amount of industrial wastewater is discharged into local industrial zone WWTPs after simple pretreatments. This type of inflow adds pressure to the normal operation of local WWTPs due to the existence of refractory organic substances (e.g., chemical synthetic dyestuff, auxiliary assistants and other chemicals). But similar to domestic sewage, the industrial effluents require biological treatment for effective removal of the organic pollutants (Orhon et al. 2009). The AS process is widely used biological treatment in all municipal-grade wastewater treatment plants due to its low operation cost (Wagner and Loy 2002). As the microorganisms in AS are the main contributors for pollutant removal, identify the functional microbes would be helpful to improve WWTP performace (Liang et al. 2014).

Many methods have been developed to investigate and characterize the microbial communities. Including cultivation based methods, traditional molecular biotechnological techniques (Zielinska et al. 2016; Yang et al. 2011) and more advanced high-throughput sequencing technology (Ye et al. 2012; Roesch et al. 2007; Qian et al. 2011; Claesson et al. 2009; Fierer et al. 2008). In one key example, pyrosequencing of 16S rRNA gene amplicons revealed a core bacterial community of 24 families and comparisons of summer and winter samples showed no significant differences in microbial community (Isazadeh et al. 2016). In another study using the Illumina HiSeq sequencing platform, 63 genera were identified as the core microbes in 13 Danish WWTPs during summer (August). Among of them, the genus *Nitrotoga* was thought to be the primary nitrite-oxidizer rather than *Nitrospirae* (Saunders et al. 2016).

However, almost all the research about the AS microbial community of WWTPs has only focused upon one single system, usually represented as a municipal sewage treatment system (Chen et al. 2016; Flowers et al. 2013; Hu et al. 2012; Lanham et al. 2013; Ma et al. 2015; Mielczarek et al. 2013; Muszynski et al. 2015; Valentin-Vargas et al. 2012; Wan et al. 2011; Wang et al. 2012; Wells et al. 2011; Xu et al. 2017; Yang et al. 2011; Zhang et al. 2012; Zielinska et al. 2016). A comprehensive comparison of municipal sewage treatment systems and industrial wastewater treatment systems is scarcely.

In this study, 5 full-scale municipal wastewater treatment systems from 3 WWTPs were selected which mainly treated domestic sewage, textile dyeing and fine chemical industry wastewater, respectively. Microbial DNA was extracted from the sludge using a liquid-nitrogen grinding pretreatment method. Metagenomic sequencing and bioinformatic analysis of the microbial communities was then carried out. The objectives were to understand the differences of the AS microbial communities and make a interpretation of their metabolic potentials as a basis for further research work.

## Materials and methods

### Sample collection

In this study, Qige WWTP, Shaoxing WWTP and Shangyu WWTP were selected for microbial community analysis. The average pollutant removal efficiency per month and influent characteristics for each of these WWTPs are summarized in Table 1. Each AS sample was sampled three times in the summer of 2015 at the same sample site (different parts of the same tank), and then mixed together. Every mixed sludge sample was centrifuged (4500×*g*, 5 min, room temperature) and the supernatant removed. Samples were stored at − 80 °C prior to DNA extraction. The sample sites and environmental parameters are also shown in Table 2.

**Table 1:** The influent and effluent water qualities of WWTPs

WWTPs (process)	BOD (mg/L)	COD (mg/L)	T-N (mg/L)	T-N removal	NH_4_^+^–N removal	Influent B/C	Influent COD/T-N	Influent COD/NH_4_^+^–N	Remarks
Qige WWTP (A/A/O)									
Influent	180–220	450–550	26.42	68.50%	94%	0.47	> 9.09	16.67	Domestic sewage treatment
Effluent	2.5–7.5	13–35	8.3						
Shangyu WWTP (A/O)									
Influent	120–160	400–450	35–40	50.00%	95.40%	0.32	11.3	12.96	Fine chemical industry wastewater
Effluent	10–15	80–92	16–21						
Shaoxing WWTP-I (A/O)									
Influent	137–160	633–661	74	42.30%	99.25%	0.23	4.9	9.3	Textile dyeing industry wastewater
Effluent	2.13–13	60–65	42.7						
Shaoxing WWTP-II (oxidation ditch)									
Influent	104–211	462–688	73.6	34.40%	97.50%	0.274	6.6	10.55	
Effluent	9.7–12.5	54–154	48.3						
Shaoxing WWTP-III (oxidation ditch)									
Influent	The same with Shaoxing			39.86%	99.25%	0.23	4.9	9.3	
Effluent	2.13–13	60–65	44.5						

WWTP-I, the first phase project; WWTP-II, the second phase project; WWTP-II, the third phase project; BOD, Biological Oxygen Demand (mg/l); COD, chemical oxygen demand (mg/l); T-N, total nitrogen concentration (mg/l); T-P, total phosphorus concentration; NH_4_^+^–N, ammonium nitrogen

**Table 2:** Description of samples collected and important WWTP parameters

Date	2015/8/15			2015/8/17		2015/8/18			
Temperature (°C)	28			32		32		32	32
WWTP name	Qige WWTP			Shangyu WWTP		Shaoxing WWTP I		Shaoxing WWTP-II	Shaoxing WWTP-III
Sampling site	Anaerobic tank	Anoxic tank	Aeration tank	Anaerobic tank	Aeration tank	Anaerobic tank	Aeration tank	Oxidation ditch	Oxidation ditch
Code	QG-Ana	QG-Ano	QG-O	SY-A	SY-O	SX-1-A	SX-I-O	SX-II (OD)	SX-III (OD)
Valid tank volume (m^3^)	5792	22,010	43,443	9640	36,730	44,640	216,691	818,670	302,000
Hydraulic retention time (h)	1	3.8	7.5	4.11	15.67	8.5	15	43.6	40.3
Sludge retention time (days)	15–18			6–14		15–22		10–17	
pH value on sample	7.5	7.4	7.21	8.18	7.92	7.49	7.62	7.3	7.9

### DNA extraction

In this study, High molecular weight community DNA was extracted by the freeze-grinding, SDS-based method and was purified using a commercialized DNA isolation kit (e.g. E.Z.N.A., Omega, Norcross, Georgia, US) (Zhang et al. 2017). Following DNA extraction, the integrity of the DNA was tested using gel electrophoresis, and concentration and purity were determined using a Qubit Fluorometer (Thermo, USA). Then 16S rRNA based sequencing and metagenomic sequencing were conducted consequently.

## 16S based high-throughput sequencing and data analysis

The V3–V4 hypervariable region of the 16S rRNA genes was amplified by primer set 340F (5′-CCTACGGGNBGCASCAG-3′) and 806R (5′-GGACTACNVGGGTATCTAAT-3′) (Fadrosh et al. 2014; Murphy et al. 2010). A 50-μL PCR reaction system was performed for each amplification using a Phusion high-fidelity PCR master mix with a HF buffer. The amplification was conducted in an XP cycler (Bioer) as follows: Initial denaturation at 98 °C for 30 s; 30 cycles at 98 °C for 15 s, 50 °C for 15 s, and 72 °C for 15 s; with a final extension at 72 °C for 1 min. Each 50 μL of PCR mixture contained 25 μL of PCR mix buffer, 3 μL of DMSO, 1 µM primer, 3 μL of each dNTP and 10 μL of genomic DNA. Nuclease free water was added up to 50 μL. The PCR products were separated by 2% agarose gel electrophoresis (6 v/cm). The bands of the expected sizes were purified using an AXYGEN gel extraction kit (AP-GX-250G, AXYGEN) following the manufacturer’s instructions. The sample DNA was sequenced using an Illumina MiSeq desktop sequencer (2 × 300 bp paired-end run, San Diego, CA) according to standard protocols. The sequence read processing was performed using QIIME (version 1.9.0) with the following quality control criteria: 1) removal of reads with ambiguous nucleotides; 2) removal of reads of less than 150 bp; 3) removal of reads containing homopolymers of ≥ 6 bp; 4) the establishment of a quality window of 50 bp with an average flowgram score of 25 (Caporaso et al. 2010). The sequences have been deposited in the NCBI Short Read Archive under accession number: SRP110572 (SRX2972725–SRX2972734).

The reads were then assigned to their corresponding samples according to their barcodes, denoised using Denoiser (Reeder and Knight 2010), clustered using uclust (Edgar 2010), and then assigned to their operational taxonomic units (OTUs) at 3% dissimilarity. The most abundant reads were selected as representatives from each OTU for de novo alignment using MUSCLE and alignment against the Greengene v13_8 database using QIIME (Edgar 2004). The species diversity, richness, and rarefaction curves were computed at 3% dissimilarity as part of the QIIME alpha diversity and beta diversity pipeline. The beta diversity was analyzed after rarefying the samples in the smallest-sized library using a step size of 100 with 100 repetitions per step. Principal-coordinate analysis (PCoA) was performed in the R environment using the vegan package. To identify the dominant/distinct genera, the genus level box plot of multiple groups was generated by STAMP through the two-sided Welch’s exact test and excluded any genus which had a low effect size of a proportions ratio (< 2) and difference between proportions (< 1).

### Library preparation, metagenomic sequencing

Illumina shotgun DNA library construction and sequencing was conducted by the Beijing Genomic Institute at Shenzhen, China. Specifically, after fragmentation, paired end fragment library in length of ∼ 170 bp was constructed. Adaptor-appended fragments were sequenced on Illumina MiSeq desktop sequencer (2 × 300 bp paired-end run, San Diego, CA) according to standard protocols.

Reads of average length of 90 bp for each end were generated. Reads were excluded from further analysis if they were shorter than 35 bp, had more than 3 ambiguous nucleotides, had 15 bp or more overlapping regions with adapter sequences, had more than 36 nucleotides with a quality value lower than 20, or were potential duplicated reads from amplification artefacts. The sequences were deposited to the Metagenomics RAST (MGRAST) sever with accession numbers PRJNA391055.

### Read assembly and gene prediction

The cleaned sequence reads were assembled into contigs using SOAP denovo (v 2.04, with settings of -d 1,-M 3, -R, -u, -F). Only contigs longer than 500 bp were used for further analysis. Open reading frames (ORFs) were predicted from contigs using MetaGeneMark (version 3.38) using default settings. The predicted ORFs longer than 100 nt were translated into protein sequences based on the NCBI translation table 11. CD-HIT (version 4.7) was then used to remove ‘redundant’ (or highly similar) sequences and to determine gene abundance and statistics.

### Function annotation

None-redundant protein sequences of the predicted genes were used to search against the NCBI NR (17 Aug. 2017), eggNOG (version 4.5) and against the KEGG (28 Jul. 2017) databases using BLASTP with the E-value cut-off of 10^−5^. The abundance of a certain COG or KEGG entry in each sample was calculated by the total number of found genes weighted by their coverage. We searched gene function of nitrate/nitrite reductase, nitric oxide reductase, nitronate monooxygenase, nitrogen fixation protein, nitrate/nitrite transporter, hydroxylamine reductase, nitrous oxide reductase, periplasmic nitrate reductase, ammonia monooxygenase, ferredoxin-nitrite reductase, nitrate reductase alpha/beta, hydroxylamine oxidoreductase, formate-dependent nitrate reductase in the eggNOG, KEGG and NCBI NR database. Then, we obtained their abundance in our samples from the search results. The abundance of KEGG function of xenobiotic biodegradation and sulfur metabolism was also obtained from KEGG search results.

## Results

Due to the presence of too much synthetic dye in the industrial wastewater treatment systems, the influent of the 3 full-scale WWTPs in Shaoxing and Shangyu WWTP usually contained a certain amount of sulfide and refractory organic pollutants. The specific organic pollutant in Shaoxing WWTP is reported as terephthalic acid (Yi et al. 2015), dyeing aids (e.g., anhydrous sodium sulfate), and acids (e.g., sulfuric acid) (Robinson et al. 2001). Comparatively, the performance of Qige WWTP had the best performance in COD, BOD and nitrogen removal which was ascribed to the better biodegradable in inflow. The highest COD removal rate was achieved in Qige-WWTP (average 91.1%), where the effluent consistently met the national Grade 1-B standard. The COD removal rate of Shangyu WWTP was the lowest (less than 77%). The T-N removal rate for industrial wastewater treatment system (Shaoxing and Shangyu WWTPs) was not higher than 50%, of which SX-II (OD) had the lowest T-N removal rate (average 34%). However, a highly stable NH_3_–N removal rate was demonstrated in all WWTPs. The detailed performance data for the summer is shown in Table 1.

### OTU analysis of sludge samples

After quality assessment, with the removal of any sequence reads of less than 150 bp, a total of approximately 342097 sequence reads were obtained in this study (Table 3). The Shannon index of municipal WWTP was shown to be higher (the three samples from the Qige WWTP ranging from 8.9256 to 9.2775) than for the textile dyeing (the four samples from the Shaoxing WWTP ranging from 6.7722 to 8.117) and fine chemical industrial wastewater treatment systems (the two samples from the Shangyu WWTP being 6.2174 to 7.1181). As shannon index was used to measure microbial biodiversity (Schloss et al. 2011). So our results is accord with the previous conclusion that the taxonomic richness of municipal AS is greater than that of industrial AS (Ibarbalz et al. 2016).

**Table 3:** OTUs number and sequencing statistical tables

Sample name	OTUs number	OTUs Seq	Coverage	Chao1	Shannon (bit)	Ace	Simpson
QG-Ana	2565	11,799	0.88	3830.2709	8.9256	4280.8225	0.9881
QG-Ano	3249	18,823	0.92	4057.4247	8.9634	4574.3833	0.9886
QG-O	3419	17,254	0.9	4516.8361	9.2775	5072.5894	0.9916
SX-I-O	2686	23,882	0.95	3621.9942	8.1170	3963.6947	0.9787
SX-I-A	2779	24,805	0.96	3318.1739	7.9507	3695.3476	0.9800
SX-II(OD)	3613	45,543	0.98	3702.4586	6.7722	4105.2816	0.8890
SX-III(OD)	2302	21,430	0.95	2975.8851	7.1415	3346.1279	0.9587
SY-A	1759	13,842	0.95	2101.0361	7.1181	2417.2464	0.9585
SY-O	2264	25,262	0.96	2879.0802	6.2174	3241.7658	0.9258

Chao1 and Shannon indexes, and sample coverage were calculated with Mothur at 97% similarity level

OTUs number, annotated operational taxonomic units number; OTUs Seq, raw operational taxonomic units sequences

### Microbial community similarity and morphologies of activated sludge

**Fig. 1:**
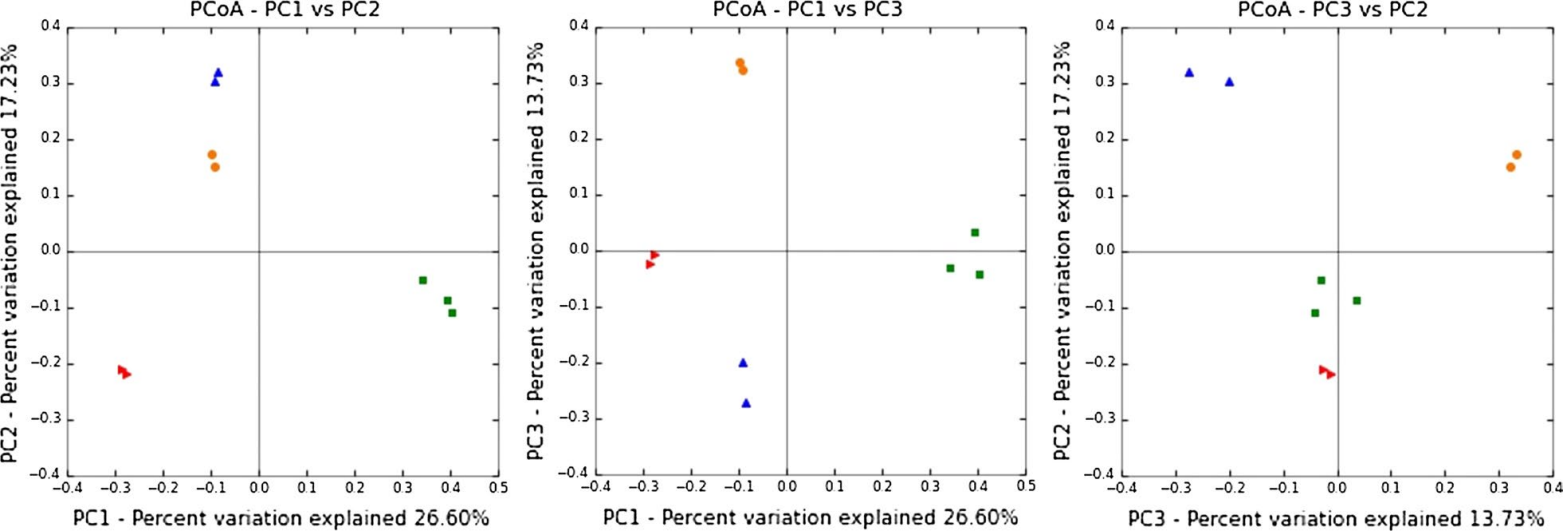
Principal coordinate analysis (PCoA) of 9 samples based on the composition of bacterial community. Group1 (red): SY(A), SY(O), Group2 (blue): SX-I(A), SX-I(O), Group3 (orange): SX-II(OD), SX-III(OD), Group4 (green): QG (Ana), QG(Ano) and QG(O)

Taxonomic assignment was based on the best achievable results of the RDP classifier with an 80% confidence threshold. PCoA analysis indicated that there was a clear delineation between groups at the taxonomic level (Fig. 1). The results showed that the microbial communities of each of the 9 samples could be clustered into 4 groups.

**Fig. 2:**
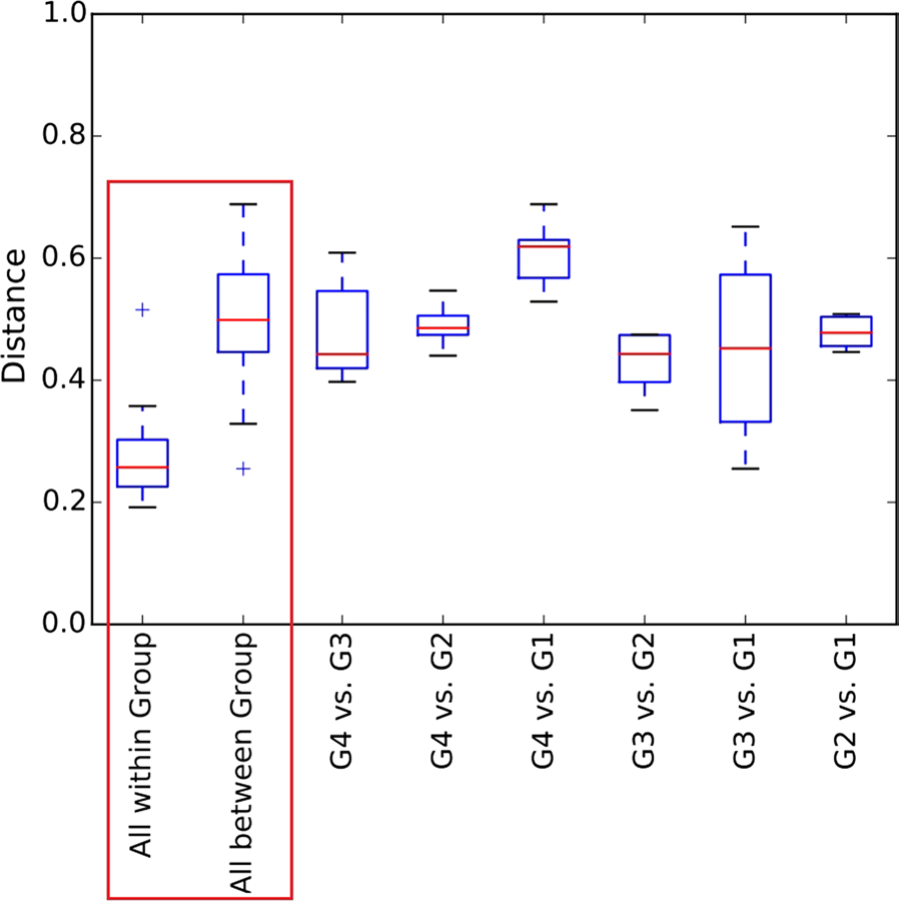
Group distance. The tests of significance were performed using a two-sided Student’s two-sample *t* test

**Fig. 3:**
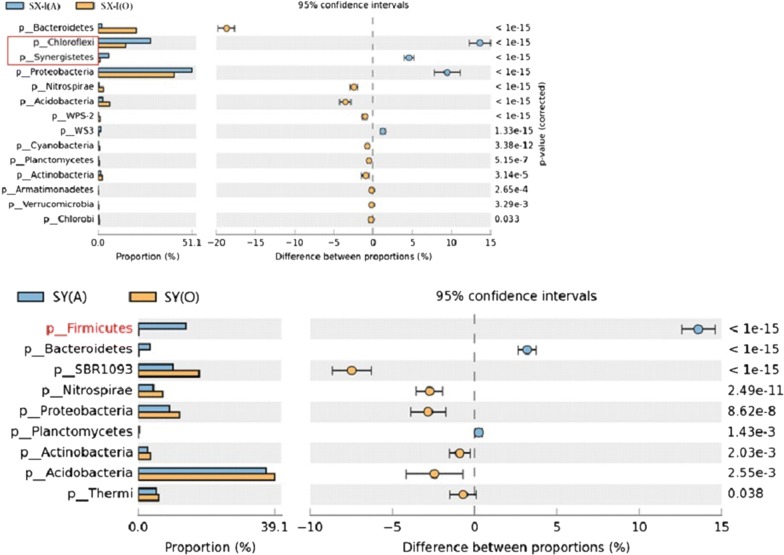
The difference of bacterial communities between two biological treatment units of A/O process system at phylum level (generated by STAMP). UP: Shaoxing WWTP-I, Down: Shangyu WWTP

The lower “within group” distance indicate that influent composition impose a clear effect on microbial community composition (Fig. 2). In another aspects, the plants using A2O or A/O process are usually built with a number of successive tanks of the same type. Besides dissolved oxygen concentration (DO), this successive tanks don’t really display any other differences. However, different and distinct microbes still can be found in the anaerobic tanks of these wastewater treatment systems (Fig. 3). So, DO is also a factor affecting microbial community composition. In this study, we found the distance between-group to be almost 2 times that of the distance within-group (Fig. 2). This implies that the effect of influent type on the microbial community is greater than the effect of dissolved oxygen.

Another factor that determines the group is process. The four samples from the Shaoxing WWTP are divided into two group samples (Group 2 and Group 3) in regard to the higher abundance of *Proteobacteria* in group 2. This might be ascribed to different processes as the two samples of group 2 were from Shaoxing WWTP-I which adopts AO processes whereas the samples of group 3 were from the carrousel oxidation ditches.

**Fig. 4:**
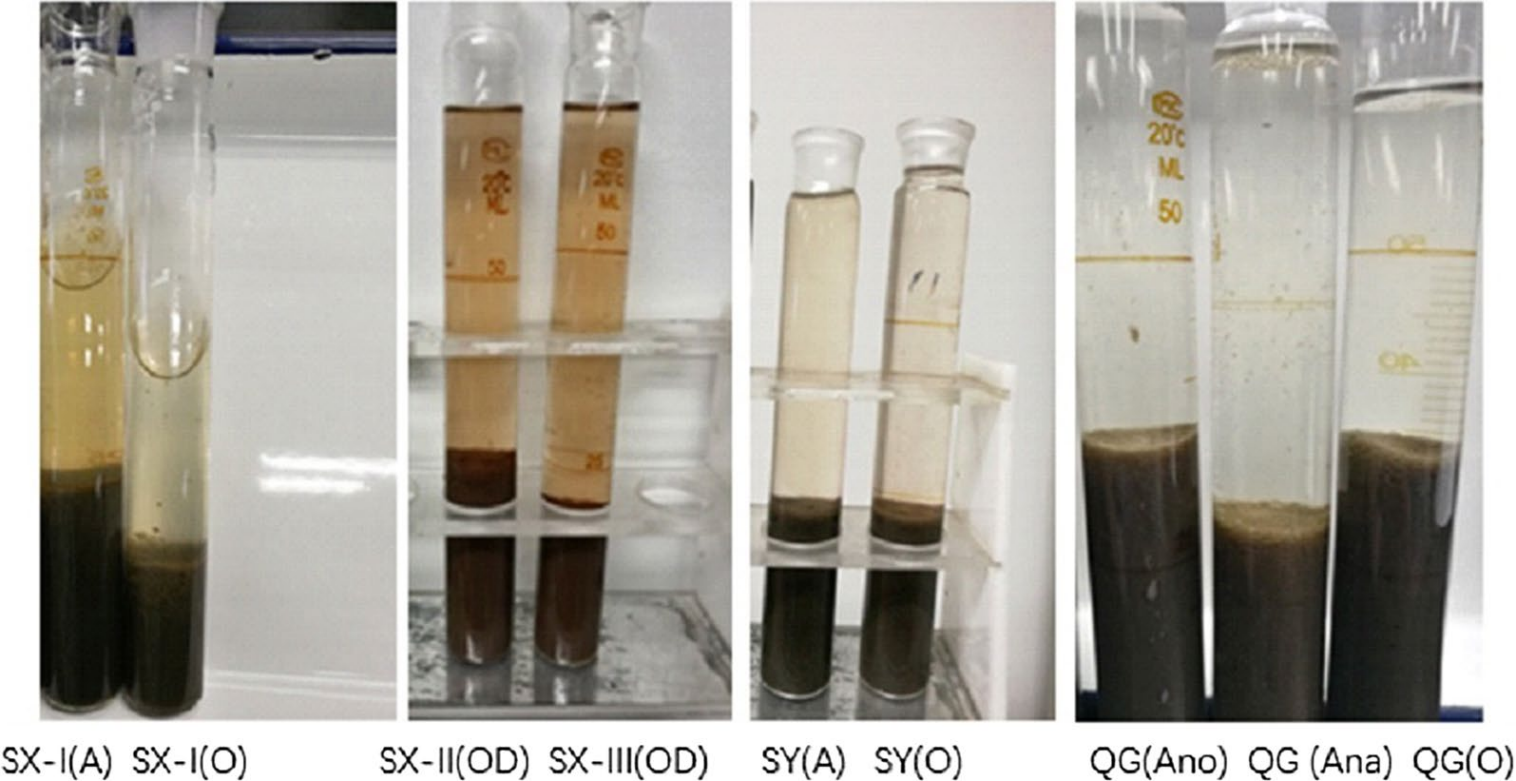
Morphologies of activated sludge samples

The morphologies of these AS were different in their colour (Fig. 4), SX-II(OD) and SX-III(OD) were reddish-brown in colour due to the presence of Fe^3+^.

### Bacterial community composition analysis

**Fig. 5:**
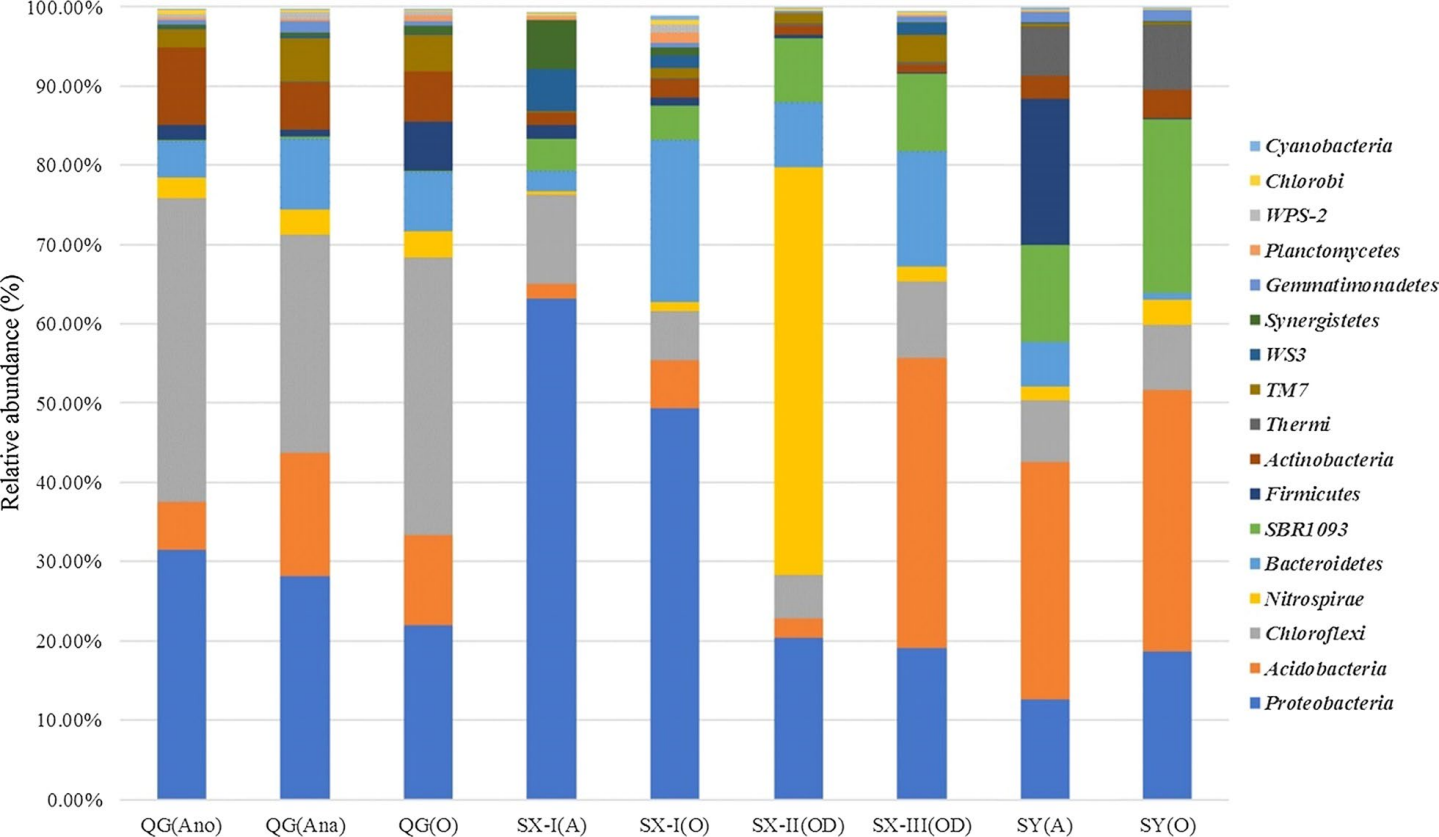
Activated sludge bacterial community composition at phylum level. *Taxa lower than 0.2% were excluded

As shown in Fig. 5, the dominant phyla in every sample included *Proteobacteria* (12.3–58.5%), *Acidobacteria* (1.8–35.1%), *Chloroflexi* (2.8–37.7%) and *Bacteroidia* (0.7–19.2%). This is consistent with previous studies on municipal WWTPs (Chen et al. 2016; Cydzik-Kwiatkowska and Zielinska 2016; Hien et al. 2011; Nielsen et al. 2010; Wan et al. 2011; Wang et al. 2012; Yang et al. 2011; Ye et al. 2012). In addition, *Actinobacteria* (0.7–6.8%), *TM7* (0.1–5.2%), *Synergistetes* (0.02–5.6%) and *Thermi* (0.03–7.89%) were also present in every sample but not always as abundant organisms (Additional file 1).

*Nitrospirae* were particularly dominant in the bacterial community composition of the oxidation ditch in Shaoxing WWTP-II, accounting for 48.68%. To the best of knowledge this is the highest content of *Nitrospirae* in any reported WWTP (Chen et al. 2016; Cydzik-Kwiatkowska and Zielinska 2016; Ma et al. 2015; Saunders et al. 2016; Xu et al. 2017; Ye et al. 2012; Zhang et al. 2012). It was also notable that the oxidation ditch of Shaoxing WWTP-III had abundant *Acidobacteria* (34.82%).

The sludge samples from Shangyu WWTP mainly consisted of *Acidobacteria, Proteobacteria,* Candidatus phylum *SBR1093, Chloroflexi* and *Thermi*. *Thermi* has been found in geothermal springs all over the world and is known to be involved in NH_4_^+^–N removal (Coman et al. 2015; Liang et al. 2016; Panda et al. 2016; Paul et al. 2016). Due to the fact that industrial wastewater is usually discharged with an amount of heat, the influent temperature could hit 40 °C in summer. Thus, an abundance of *Thermi* should be associated with the typical influent of the Shangyu WWTP where high temperatures and appropriate organic substances in the influent have created an ideal external environment.

To identify the AS microbial community’s composition in detail, the reads were further analyzed and assigned to a genus level (Additional file 2). Each WWTP could be characterized by its set of the most abundant or distinct genera. Combining this data with the WWTP performance in the summer of 2015 (Table 1), we will now discuss the dominant and/or distinct genera and parse their function related to pollutant removal.

## Discussion

**Fig. 6:**
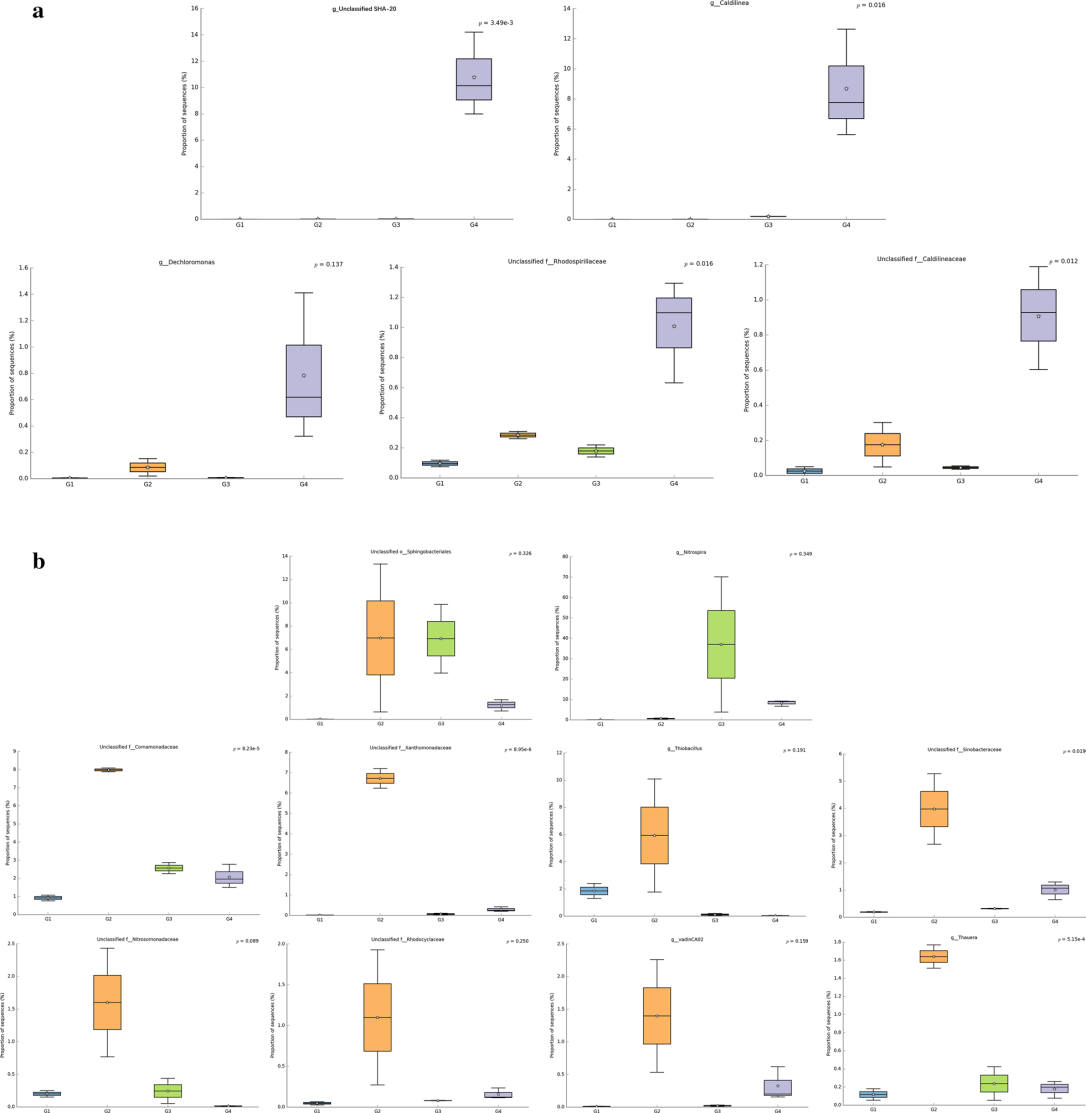
The dominant and distinct genera in Qige WWTP (**a**), Shaoxing WWTP (**b**). G1: Group1, G2: Group2, G3: Group3, G4: Group4

The dominant and distinct genera in the Qige WWTP included unclassified *SHA*-*20*, *Caldilinea*, *Dechloromonas*, and unclassified genera from *Rhodospirilaceae* and *Caldilineaceae* (Fig. 6a). Putting aside the unclassified genera, of the known genera the *Caldilinea* have been reported to include some filamentous species and play a role in forming flocs of AS in a wide range of WWTPs (Yoon et al. 2010). *Caldilinea* were represented from 8.16 to 10.83%. *Dechloromonas* accounted for 1.67% in the anoxic tank. This genus is capable of reducing perchlorate and chlorate, which is associated with nitrate reductase (Achenbach et al. 2001). Moreover, *Dechloromonas* is frequently reported as a phosphate accumulating organism in enhanced biological phosphorus removal reactors (Liu et al. 2005). As the AS in the Qige WWTP presented higher biodiversity, but most of the known genera represented lower than 1%, so the potential functions of these genera require further investigation. Meanwhile, the functional redundancy contained in such a mix should enable the system to resist environmental perturbations and maintain stable COD, P, and N removal where some of the core genera can be replaced by others having the same functional role in AS. In this case there may be no need for some dominant genera to be present (Allison and Martiny 2008; Bradley and Pollard 2017; Johnson et al. 2015).

**Fig. 7:**
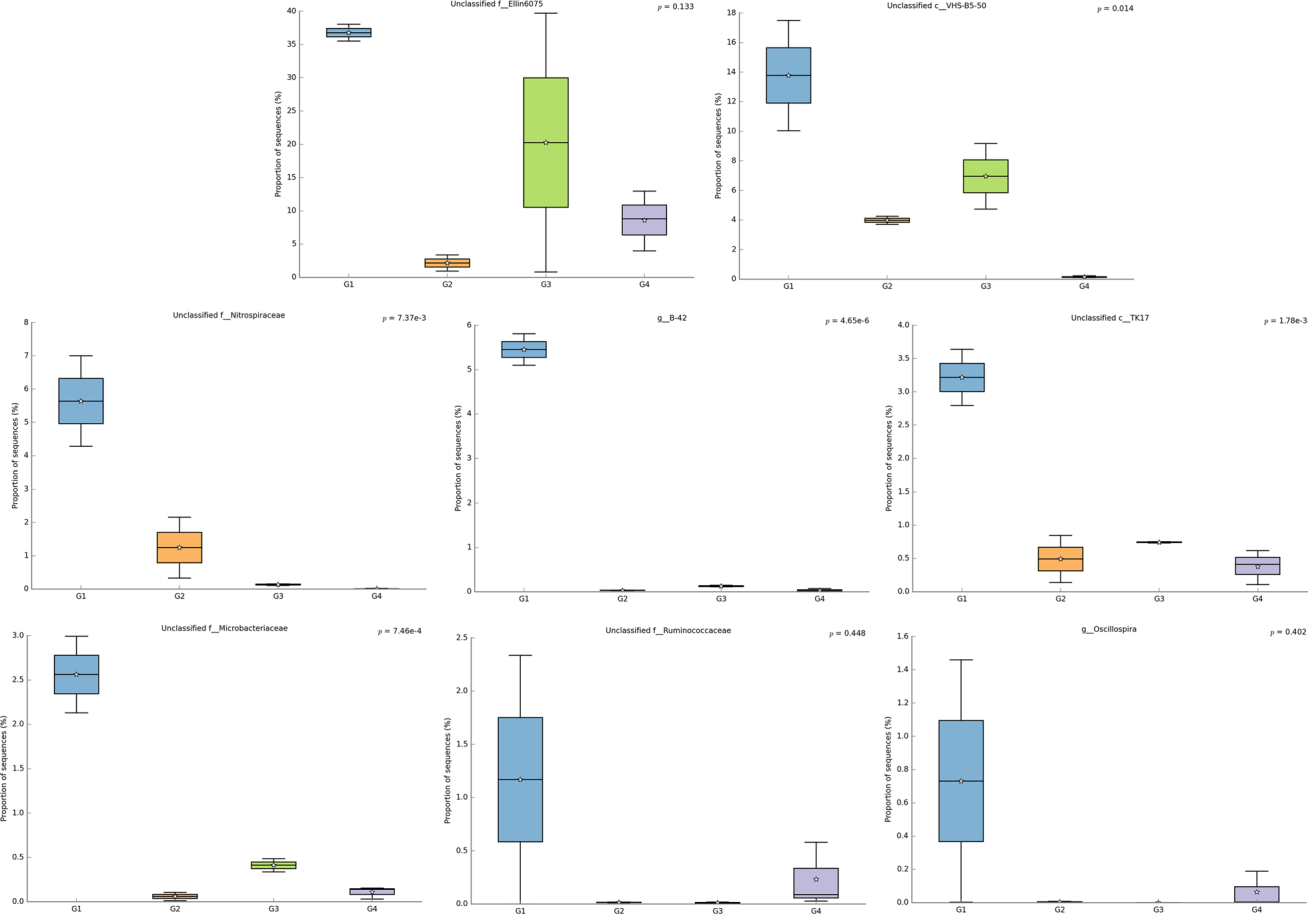
The dominant and distinct genera Shangyu WWTP. G1: Group1, G2: Group2, G3: Group3, G4: Group4

As mentioned in section 3.2, the WWTPs of Shaoxing are divided into two groups. As a whole, the dominant and distinct genus in the WWTPs of Shaoxing is an unclassified genus from *Sphingobacteriales*. The dominant and distinct genera of Group 2 included *Thiobacillus*, *VadinCA02*, *Thauera* and unclassified genera from *Comamonadacae*, *Xanthomonadacae*, *Sinobacteraceae*, *Nitrosomonadaceae* and *Rhodocyclaceae*. The dominant and distinct genus of Group 3 is *Nitrospira*. The details can be seen in Fig. 6b. *Sphingobacteriales* has been reported to function as a BPA (bisphenol A) biodegradation bacterium (Oshiman et al. 2007). It could function as organic dyestuff degrader in an aerobic tank and provide monomer or oligomer carbon sources for *Thiobacillus* which can use non-organic carbon sources, such as ${\text{CO}}_{3}^{{^{ 2- } }}$ and ${\text{HCO}}_{3}^{{^{ - } }}$. *Thiobacillus* and *Thauera* are all involved in aromatic compound degradation and denitrification conditions (Mao et al. 2010; Thomsen et al. 2007; Foss and Harder 1998; Shinoda et al. 2004; Zhang et al. 2015; Fernandez et al. 2009; Vishnivetskaya et al. 2013). Some strains from *Comamonadaceae* not only use phenol and their derivatives as energy sources but also act as a potential aerobic denitrifiers that directly transform ammonia into nitrogen under aerobic conditions (Ni et al. 2013). *Xanthomonadaceae* are obligate aerobes. Some of them have been directly or indirectly related to petroleum hydrocarbon degradation (Martinez-Lavanchy et al. 2015). *Sinobacteraceae* prefer to utilize aliphatic, aromatic hydrocarbon compounds and small organic acids for degradation (Gutierrez et al. 2013). They play crucial roles in the degradation of organic compounds and form the floc structure of AS (Khan et al. 2002; Shchegolkova et al. 2016). *Nitrosomonadaceae* is a well-known ammonia oxidizer (Black et al. 2017). *Rhodocyclaceae* and *Xanthomonadaceae* are reported to participate the simultaneous biodegradation of phenol and ammonia oxidation (Fitzgerald et al. 2015; Liu et al. 2017).

Due to the high dissolved oxygen concentration (3.0–5.0 mg/L) in an oxidation ditch, the NH_3_–N removal rate is high. However, the nitrogen removal rate at Shaoxing WWTP II and III was lower than in others systems. This poor denitrification ability could be due to high nitrate accumulation. This might also account for the high abundant ammonia oxidizers and nitrifiers like *Nitrosomonadaceae* and *Nitrospira* (as above and see Additional file 2) (Daims et al. 2015). The reasons for the nitrate accumulation at Shaoxing WWTP II were the low COD/N rate and the oxidation ditch process itself. Without a carbon source, the system cannot perform denitrification. Without anaerobic or anoxic units, the denitrification ability of ordinary denitrification bacteria would be inhibited regardless of the high dissolved oxygen concentration in the treatment system. Adding an enrichment culture of *Thiosphaera, Pseudomonas, Alcaligenes, Paracoccus, Bacillus* and *Zoogloeal* would be an ideal protocol for resolving this problem as these bacteria function as aerobic denitrifiers (Chen and Ni 2011; Ji et al. 2015).

The dominant/distinct genera of Shangyu WWTP are *B*-*42* from the family *Trueperaceae*, *Oscillospira,* an unclassified genus from family *Ellin6075, Nitrospiraceae, Microbacteriaceae, Ruminococcaceae*, plus unclassified genus from class *Vh5*-*B5*-*50* and *TK17* (Fig. 7). Except for the most two distinct and dominant genera, unclassified genus from the family *Ellin6075* order *RB41* in *Chloracidobacterium* and unclassified genus from family *VHs*-*B5*-*50,* the functions of which are still unknown to us. All the others microbes function in refractory organic degradation and nitrogen removal. Such as, *Oscillspira* was reported as involved in ferric reduction and/or high concentration dimethyl sulfide (DMS) degradation (Ye et al. 2016). *Nitrospiraceae* is known for its autotrophic nitrite oxidation and can be found in thermal underwater environments (Marks et al. 2012). *Microbacteriaceae* was reported that can grow in thermal underwater ecosystems and play important roles in denitrification (Sharma et al. 2017; Tomasek et al. 2017). And some *Microbacteriaceae* strains can even grow in a heavy metal contaminated environments (Corretto et al. 2017). *Ruminococcaceae* are able to produce mixed byproducts containing H_2_, ethanol, acetate and degrade Methyl tert-Butyl Ether (MTBE) (Liu et al. 2016; Veeravalli et al. 2017). Although we also know nothing about genus *B*-*42*, but as for *Trueperaceae*, it has been reported as a hydrocarbon-bearing microorganism where some strains from *Trueperaceae* have been seen to have the ability to grow under multiple extreme conditions such as high alkalinity, moderately salinity, high temperatures, and are even found t o be present and remarkably resistant to ionizing radiation (Corretto et al. 2017; Qian et al. 2017). Additionally, *Thiobacillus* was also found (1.49% in the anaerobic tank and 3.02% in the oxic tank). This result was perhaps due to the higher presence of chemical industrial wastewater influent at this plant.

Although many municipal and industrial WWTP have been analyzed using next generation sequencing (NGS). However, previous work about AS microbial community composition, were all done by extracting DNA with normal commercial isolation kit without properly considering the bias induced by the DNA extraction method. However, this bias had been clearly reported (Saunders et al. 2016). Furthermore, all the previous studies took aeration tank samples as if they were automatically representative of the whole WWTP and half of them used traditional molecular biotechnology (Chonova et al. 2016; Flowers et al. 2013; Hug et al. 2005; Jiang et al. 2016; Ju et al. 2014; Kim 2013; Muszynski et al. 2015; Tuncal et al. 2009; Wang et al. 2016). Again, most of the known research work only had its focus on municipal sewage treatment systems (Chen et al. 2016; Cydzik-Kwiatkowska and Zielinska 2016; Ding et al. 2017; Isazadeh et al. 2016; Jiang et al. 2016; Jin et al. 2011; Ju et al. 2014; Lu et al. 2014; McLellan et al. 2010; Muszynski et al. 2015; Wan et al. 2011; Wang et al. 2016; Xu et al. 2017; Zhang et al. 2012; Zielinska et al. 2016). To make more comprehensive analysis on the AS microbial community of WWTP, samples were taken from every biological treatment unit of the specific industrial WWTPs with increased industrial inflow, not just the aeration tanks.

Kinetics of growth and decay are typically too slow to expect changes in community composition between AS from connected tanks of the same WWTP [e.g. SX-I(A)/SX-I(O), SY(A)/SY(O), QG(Ana)/QG(Ano)/QG(O)]. To some extent, it is reasonable to consider the oxic tank AS sample as representative of A/O and A2/O process systems. The similarity analysis gives a clear empirical test for this positive association that has not been previously provided.

As presented by the research from Aalborg university, the concept of core community is useful to identify putatively important organisms (Saunders et al. 2016). Compare with domestic sewage treatment system, distinct genera was more easier can be found in industrial wastewater treatment systems. The large scale of the textile dyeing industrial influents broadens the ecological amplitude of these aromatic compound degradation and denitrification bacteria which adapted to oligotrophic wastewater with low COD loading and perform the biodegradation of hazardous organic pollutants. The key functional genera of textile-dyeing industrial wastewater system would be *Nitrospira, Sphingobacteriales, Thiobacillus, Sinobacteraceae* and *Comamonadaceae* becomes reasonable.

The functional profile of the representative of four groups AS samples [QG(O), SX-I(O) SX-II(OD) and SY(O)] were analyzed through metagenomic sequencing. Most of the genes were successfully classified into hierarchical metabolic categories using unscaled Manhattan variance distances and these are presented in a triplex hierarchical table (Additional file 3). The abundance of inferred genes from KEGG were generally lower than 1% (Additional file 4), except for genes encoding transporters (ranging from 4.4 to 6.4%) and ABC transporters (ranging from 2.7 to 4.7%). Compared with the obvious bacterial community differences between groups, most of the effects of gene function in level 3 of KEGG were equally distributed within each of the groups. These inferences of metabolic functional genes did not show a consistent trend with the variation of the AS microbial community. In this way, a slow metabolic turnover between plants can be conceived. The reason why significant difference in microbial community but not in functional traits likely due to DNA nucleotide sequences was not as equivocal as phenotypic characters. All cells must produce proteins for their survival and eventual replication through transcribe the specific DNA into a single strand of messenger RNA, then this nucleotide sequence in mRNA is translated into a specific amino acid sequence of the protein. However, except for the essential functions in cells, most of gene expression were not conserved. To investigate their metabolic difference, functional profiles of these microbial consortia should be investigated through metatransgenomic analysis.

Considering excessive quantities of industrial inflow, we further investigated differences in xenobiotic biodegradation at the level 3 of KEEG. Comparatively, a distinctly high prediction of functional genes related to nitrotoluene degradation could be found in SX-II(OD). However, the proportion was still lower than 0.5% and presented with a high *P* value (0.573), which could be considered a non-significant difference. Difference were also evident in the individual genes related to nitrogen removal between samples based on the KEGG Orthology database with an acceptable P value (lower than 0.05). However, the proportion was even less than 0.07% at the highest point. So, the bacterial communities from different wastewater treatment systems exhibited only minor metabolic potential differences.

## Additional files

**Additional file 1.** The relative abundance of microbial communities at the phyla level.

**Additional file 2.** The relative abundance of microbial communities at the genus level.

**Additional file 3.** KEGG based triplex hierarchical metabolic pathway composition statistic table.

**Additional file 4.** KEGG based functional enzyme encoding gene composition statistic table.

## Abbreviations

AS: activated sludge
WWTP: wastewater treatment plant
PCR: polymerase chain reaction
SDS: sodium dodecyl sulfate
BOD: biochemical oxygen demand
COD: chemical oxygen demand
T-N: total Kjeldhal nitrogen
NH_3_–N: ammonia nitrogen
T-P: total phosphorus
DMSO: dimethyl sulfoxide
OTUs: operational taxonomic units
RDP: ribosomal database project
ACE: abundance based coverage estimator
A2O: anaerobic-anoxic–oxic process
OD: oxidation ditch process
AO: anaerobic-oxic process
PCoA: principal coordinate analysis
BPA: bisphenol A
